# The Correlation Between Peroxisome Proliferator-Activated Receptor Alpha and Gamma Polymorphisms and Acute Coronary Syndrome

**DOI:** 10.7759/cureus.26147

**Published:** 2022-06-21

**Authors:** Aykut Kemanci, Tarik Goren, Mehmet Uluturk, Atakan Yilmaz, Ramazan Sabirli, Mert Ozen, Murat Seyit, Alten Oskay, Aylin Koseler, Ibrahim Turkcuer

**Affiliations:** 1 Emergency Medicine, Tavsanli Doc. Dr. Mustafa Kalemli State Hospital, Kutahya, TUR; 2 Emergency Department, Medical Faculty, Pamukkale University, Deni̇zli̇, TUR; 3 Emergency Department, Medical Faculty, Pamukkale University, Denizli, TUR; 4 Emergency Department, Bakircay University Faculty of Medicine Cigli Training and Research Hospital, Izmir, TUR; 5 Biophysics Department, Medical Faculty, Pamukkale University, Denizli, TUR

**Keywords:** mutation, polymorphism, coronary heart disease, acute coronary syndrome, ppar gamma, ppar alpha

## Abstract

Objective: This study aims to evaluate the relationship between peroxisome proliferator-activated receptor (PPAR) alpha and gamma gene polymorphisms and acute coronary syndrome (ACS) clinically.

Subject and methods: Peripheral blood samples were collected from a total of 200 people, including 100 acute coronary syndrome patients and 100 controls aged 19 to 93 years, admitted to the Pamukkale University Emergency Medicine Department. The healthy volunteers had no known chronic or acute diseases, no history of drug use, and no recent history of coronary artery disease (CAD). PPAR alpha L162V and PPAR gamma C161T gene polymorphic regions were detected using DNA sequencing analyses. In addition, data collected from the hemogram and biochemical parameters and comorbidities of the patients were statistically analyzed.

Results: PPAR gamma C161T polymorphisms were compared between groups. The CT heterozygous rate in the patient group (74%) was higher than in the control group (7%). The T allele was more common in the patient group (0.37) compared to the control group (0.03). When PPAR alpha L162V polymorphism was compared, VV homozygous individuals were %19 in the patient group and none in the control group. The V allele was found to be statistically higher in patients with ACS (p<0.01).

Conclusion: The findings revealed that elevated PPAR alpha L162V and PPAR gamma C161T gene polymorphisms were associated with a progressive risk of ACS.

## Introduction

Coronary artery disease (CAD) is defined as impaired blood flow to the heart due to accumulating atherosclerotic plaque within the coronary arteries [1]. The definition of acute coronary syndrome (ACS) is used for patients with confirmed or suspected myocardial infarction (MI) [2].

Research on multifactorial causes of coronary heart disease (CHD) is increasing. For example, for acute MI, the primary underlying cause is atherosclerosis, but thrombosis has been identified in almost all cases [3]. Epidemiologic studies have shown that some factors such as age, family history of CAD, suboptimal diet, tobacco use, high BMI, high blood pressure, high plasma glucose, higher intake of red meat and high-fat dairy products, physical inactivity, and psychological factors may contribute to CAD. Being physically active, not smoking, having normal blood pressure, blood glucose levels, and total cholesterol levels, being at a normal weight, and eating a healthy diet are all promoted as ideal cardiovascular health metrics [4].

Atherosclerosis is a chronic, inflammatory, fibroproliferative disease affecting medium and large arteries with lipid deposition [5]. Hyperlipoproteinemia and endothelial cell dysfunction occur in the formation of atherosclerotic plaque. Atherosclerosis is mostly associated with multifactorial and polygenic disorders rather than single-gene defects. Studies have determined that familial hypercholesterolemia and familial apolipoprotein B-100 diseases are associated with low-density lipoprotein (LDL) receptor anomalies [6]. Similarly, low high-density lipoprotein (HDL)-associated single gene defects were detected, including ABC transport defects and apolipoprotein A-1 deficiency. Also, it has been determined that hypertension is associated with 11β-hydroxylase anomalies and mineralocorticoid receptor defects [6].

Atherosclerosis plays a critical role in the development of coronary heart disease and is affected by many environmental and genetic factors. Peroxisome proliferator-activated receptors (PPARs) regulate carbohydrate and lipid metabolism. The PPAR family includes three proteins known as alpha, beta/delta, and gamma. The PPAR family regulates the migration of leukocytes into endothelial cells, controls lipid hemostasis and the inflammatory response of monocytes and macrophages, and regulates the production of inflammatory cytokines by smooth muscle cells [7]. In addition to their effects on glucose and lipid metabolism, PPARs also have many other biological functions. For example, PPAR gamma plays a crucial role in smooth muscle cell growth, free radical generation, and suppressing inflammation. Furthermore, PPARs have been found to be expressed in macrophage foam cells and atherosclerotic lesions, thus suggesting that PPAR gamma may influence atherosclerogenic processes [8].

PPAR alpha has the most metabolic activity among the PPARs. It plays a role in major metabolic changes such as improvement in metabolic syndrome and triglyceride metabolism [9]. PPAR alpha has been shown to be expressed in cell types involved in the atherosclerosis process. This process occurs when PPAR alpha affects blood lipid parameters independently from the cellular level or by contributing to them. PPAR alpha activation reduces macrophage infiltration into atherosclerotic plaque [10]. PPAR alpha activation plays an important role in the inhibition of immune inflammation with different mechanisms, such as migration into the subendothelium, mononuclear cell adhesion, production of proinflammatory cytokines (VCAM-1, IL-1β, IL-6, IL-8), and the decrease of proinflammatory activity in the endothelium [11,12].

More than a dozen genetic variants have been identified that affect the transcriptional activity of human PPAR alpha. One of the most common PPAR alpha polymorphisms reported to date and associated with changes in lipid metabolism is L162V [13]. PPAR beta/delta gene variants show little or no association with CAD [14]. Currently, two PPAR gamma polymorphisms have been identified; these are P12A and C161T. There are studies showing that the PPAR gamma C161T polymorphism may be associated with coronary heart disease [14].

PPAR alpha and gamma are expressed in macrophage foam cells and atherosclerotic lesions and play a role in the pathogenesis of atherosclerosis [15]. In this study, we aimed to evaluate the relationship between PPAR alpha and gamma gene polymorphism and acute coronary syndrome.

## Materials and methods

Statement of ethics

Ethical approval was granted by the Pamukkale University Non-Interventional Clinical Research Ethics Committee on September 13, 2019, and numbered 60116787-020/63067.

Study population

The research was conducted between August 2019 and December 2020 on patients who were admitted to the emergency department (ED) with symptoms and diagnosed with ACS. ACS is defined as ST-segment elevation MI, non-ST-elevation myocardial infarction, and unstable angina.

**Figure 1:**
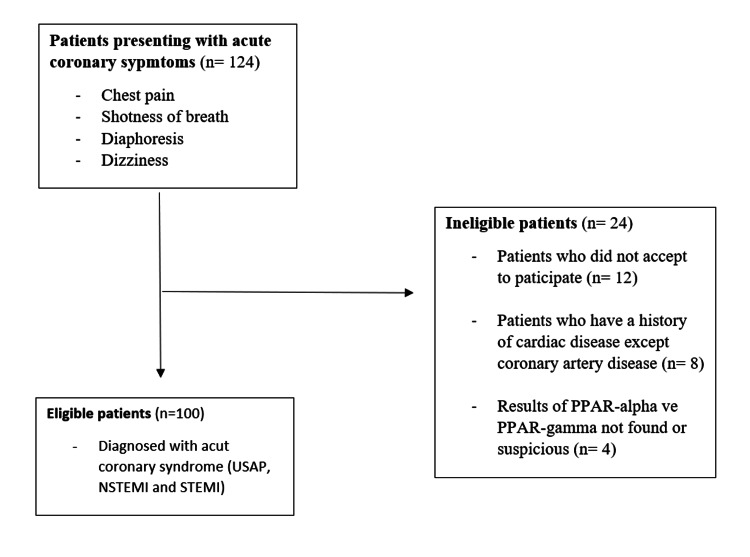
Flow diagram

Informed consent was obtained from a total of 200 individuals, 100 controls, and 100 patients, aged 18-93 years, in accordance with the declaration of Helsinki (Figure 1). This study included 100 patients and 100 healthy volunteers without symptoms as a control group. The healthy volunteers had no known chronic or acute disease, no history of drug use, and no recent history of CAD. CAD was defined as myocardial infarction, stable and unstable angina, prior coronary arterial revascularization, or >50% stenosis of a major epicardial coronary artery based on angiography.

Exclusion criteria

The exclusion criteria for the patient group in this study consisted of a history of acute pulmonary embolism or deep venous thrombosis; a diagnosis of kidney, liver, or heart failure; a history of chronic inflammatory disease; and pregnancy.

Data collection

Demographic data, medical history, vital findings (fever, blood pressure, sPO2, respiratory rate), laboratory findings (complete blood count; blood urea nitrogen (BUN), creatinine, total bilirubin, liver enzymes [aspartate aminotransferase (AST), alanine aminotransferase (ALT)], C-reactive protein (CRP), D-dimer, creatine kinase muscle-brain (CK-MB), hsTnT, and blood gas analysis parameters), total cholesterol, HDL, LDL, VLDL, triglyceride, Na, K, and comorbid diseases were included in the data set.

Genotyping

Blood samples were collected in EDTA vacutainers, and DNA was extracted from peripheral blood using the standard phenol-chloroform procedure. Genotyping of the C161T polymorphism at exon 6 of the PPAR gamma gene was performed by polymerase chain reaction (PCR). The forward primer was 5'-CAA GAC AAC CTG CTA CAA GC-3' and the reverse primer was 5'-TCC TTG TAG ATC TCC TGC AG-3' [16]. The identification of polymorphic variants of the L162V polymorphism of the PPAR alpha gene was done as described previously in the literature [17]. The forward primer was 5'- GACTCAAGCTGGTGTATGACAAGT-3' and the reverse primer was 5'- CGTTGTGTGACATCCCGACAGAAT-3. The determination of PCR products was first run on agarose gels and visualized by ethidium bromide staining and subsequent UV transillumination. Then genotyping of sequence allele marker sites was performed using the capillary electrophoresis system (ABI 3730 DNA to Analyzer, Applied Biosystems, Life Technologies Corporation, CA).

Statistical analysis

All statistical analyses were performed using SPSS 25.0 (IBM SPSS Statistics 25 software, Armonk, NY: IBM Corp.) software. Continuous variables were defined by the mean ± standard deviation, and categorical variables were defined by number and percent. The normal distribution was determined using the Kolmogorov-Smirnov and Shapiro-Wilk tests. For independent group comparisons, the Mann Whitney U test and Kruskal Wallis variance analysis (post hoc: Mann Whitney U test with Bonferroni Correction) were used when parametric test assumptions were not provided for continuous variables. The chi-square test was used for categorical variables. Logistic regression analysis was used to determine the risk factors for acute coronary syndrome. Statistical significance was determined as p≤0.05.

## Results

The average age of the patients with acute coronary syndrome participating in the study was 64 years. The average age of individuals in the control group was 62.2 years. While 75% of the patients in the case group were male and 25% were female, 54% of those in the control group were male and 46% were female.

When the patient and control groups were compared according to the presence of additional diseases, there was no statistically significant difference between the control and patient groups in terms of diabetes mellitus and hyperlipidemia. Hypertension was detected at a rate of 17% in the control group and 38% in the acute coronary syndrome group, which is statistically significant (p=0.002).

When the cardiac marker and hemogram values were compared between the patient and control groups, the CK-MB, troponin T, and WBC values of the patients were higher than those of the control group. In addition, in the comparison of the lipid profiles between the patient and control groups, the mean HDL value of the patients was found to be lower than the control group (Table 1).

**Table 1:** Comparison of hemogram, cardiac marker, and lipid values IQR: interquartile range; CK-MB: creatine kinase muscle-brain; Hs-cTnT: high-sensitive cardiac troponin T; WBC: white blood cell; Hgb: hemoglobin; Plt: thrombocyte; VLDL: very-low density lipoprotein; LDL: low-density lipoprotein; HDL: high-density lipoprotein; CRP: C-reactive protein; ALT: alanine aminotransferase; AST: aspartate aminotransferase; LDH: lactate dehydrogenase *p-values are derived from Mann Whitney U-test.

Parameters	Case (n=100)	Control (n=100)	p-values*
	Median (IQR)	Median (IQR)	
CK-MB	3.39 (8.01)	2.25 (2.43)	<0.001
Hs-cTnT	36.25 (94.3)	7.00 (5.0)	<0.001
WBC	9.940 (4.4)	8.54 (4.0)	0.006
Hgb	13.70 (2.9)	13.00 (2.1)	0.680
Plt	230.00 (96)	246.00 (88)	0.055
LDL	101.00 (43)	100.50 (35)	0.574
HDL	39.00 (10)	43.00 (18)	<0.001
Cholesterol	169.00 (39)	171.00 (29)	0.212
Triglyceride	116.50 (76)	146.00 (67)	0.062
VLDL	23.50 (15)	28.00 (13)	0.064
CRP	2.48 (6.90)	3.55 (6.08)	0.436
ALT	16.00 (11)	16.50 (12)	0.925
AST	22.00 (14)	20.50 (9)	0.172
LDH	304.00 (68)	239.00 (54)	<0.001

PPAR gamma C161T and PPAR alpha L162V polymorphism rates were compared between groups. The PPAR gamma C161T genotype C/C genotype (26%) was lower in the patient group than in the control group (93%), and the C/T genotype (74%) in the patient group was higher than the control group (7%; p<0.001) (Table 2). In the patient group, the PPAR-alpha L162V polymorphism and L/L genotype ratio (58%) were lower than in the control group (73%). The L/V genotype ratio in the patient group (23%) was statistically higher than in the control group (27%). While the V/V genotype ratio was 19% in the patient group, this polymorphism was not observed in the control group (p<0.001) (Table 2).

**Table 2:** PPAR-gamma C161T and PPAR-alpha V162T genotypes between groups *p-values are derived from the chi-square test

Gene	Case					Control				p-values*
	Genotype	n	%	Allele frequency		n	%	Allele frequency		
PPAR-gamma C161T	C/C	26	26	C	0.63	93	93	C	0.97	<0.001
	C/T	74	74	T	0.37	7	7	T	0.03	
	T/T	-	-			-	-			
PPAR-alpha L162V	L/L	58	58	L	0.70	73	73	L	0.87	<0.001
	L/V	23	23	V	0.30	27	27	V	0.13	
	V/V	19	19			-	-			

The PPAR gamma C161T polymorphism was used to compare hemogram and biochemical values. The average glucose, creatine, LDH, CK-MB, troponin, and WBC values in C/C homozygous individuals were found to be statistically lower than the average in C/T heterozygous individuals (Table 3).

**Table 3:** Hemogram and biochemical values according to PPAR-gamma C161T genotypes IQR: interquartile range; ALT: alanine aminotransferase; AST: aspartate aminotransferase; LDH: lactate dehydrogenase; CRP: C-reactive protein; CK-MB: creatine kinase muscle-brain; Hs-cTnT: high-sensitive cardiac troponin T; WBC: white blood cell; Hgb: hemoglobin

Parameters	PPAR-gamma C161T		p-values*
	C/C	C/T	
	Median (IQR)	Median (IQR)	
Glucose	109.00 (46)	131 (68)	<0.001
Creatine	0.82 (0.38)	0.97 (0.31)	<0.001
ALT	16.00 (11)	17.12 (12)	0.660
AST	21.00 (10)	22 (16)	0.515
LDH	244.00 (75)	307 (73)	0.002
CRP	3.10 (6.90)	3.48 (6.09)	0.996
CK-MB	2.68 (2.29)	3.02 (6.49)	0.007
Hs-cTnT	8.00 (8.00)	34.6 (92.60)	<0.001
WBC	8.80 (4.6)	9.87 (3.8)	0.011
Hgb	9.30 (2.2)	13.6 (2.7)	0.496

Hemogram and biochemical values were compared according to PPAR gamma C161T genotypes. The mean HDL value in C/C homozygous individuals (44.71 ± 13.71) was statistically higher than the average in C/T heterozygous individuals (39.68 ± 10.60). No statistically significant differences were found between other lipid parameters in PPAR gamma C161T genotypes (Table 4).

**Table 4:** Lipid parameters according to PPAR-gamma C161T genotypes IQR: interquartile range; LDL: low-density lipoprotein; HDL: high-density lipoprotein; VLDL: very-low-density lipoprotein *p-values are derived from Mann Whitney U-test.

Parameters	PPAR-gamma C161T		p-values*
	C/C	C/T	
	Median (IQR)	Median (IQR)	
LDL	99.00 (35)	101.00 (43)	0.269
HDL	43.00 (16)	39.00 (9)	0.002
Cholesterol	171.00 (34)	171.00 (42)	0.593
Triglyceride	146.00 (68)	120.00 (75)	0.31
VLDL	28.00 (13)	24.00 (15)	0.302

The mean CRP of V/V homozygous individuals (4.55 ± 7.09) was higher than that of L/V heterozygous individuals (4.55 ± 7.09) and L/L homozygous individuals (9.19 ± 18.77; p = 0.026). The mean of CK-MB was found to be higher in V/V homozygous individuals (17.04 ± 32.23) than in L/V heterozygous (7.70 ± 19.02) and L/L homozygous individuals (8.03 ± 25.47; p = 0.011). The mean troponin of V/V homozygotes (174.83 ± 303.55) was higher than in L/V heterozygous (61.23 ± 169.07) and L/L homozygous individuals (58.01 ± 155.94; p = 0.002). No statistically significant differences were found for the other hemogram and biochemical parameters (Table 5).

**Table 5:** Hemogram and biochemical values according to PPAR-alpha L162V genotypes IQR: interquartile range; ALT: alanine aminotransferase; AST: aspartate aminotransferase; LDH: lactate dehydrogenase; CRP: C-reactive protein; CK-MB: creatine kinase muscle-brain; Hs-cTnT: high-sensitive cardiac troponin T; WBC: white blood cell; Hgb: hemoglobin * p-values are derived from the Kruskal-Wallis test

Parameters	PPAR-alfa L162V			p-values*
	L/L	L/V	V/V	
	Median (IQR)	Median (IQR)	Median (IQR)	
Glucose	117.00 (61)	112.00 (39)	132.00 (57)	0.178
Creatine	0.89 (0.40)	0.92 (0.42)	0.88 (-0.29)	0.840
ALT	17.00 (11)	15.00 (10)	16.00 (12)	0.830
AST	21.00 (11)	19.00 (10)	25.00 (14)	0.228
LDH	265.00 (101)	240.50 (82)	245.00 (92)	0.227
CRP	4.10 (7.64)	1.77 (3.34)	3.18 (7.26)	0.026
CK-MB	2.70 (2.41)	2.72 (3.23)	5.60 (12.28)	0.011
Hs-cTnT	9.00 (29.06)	7.50 (13.58)	45.64 (118.00)	0.002
WBC	9.17 (5.2)	9.14 (4.1)	9.87 (3.1)	0.645
Hgb	13.30 (2.4)	13.40 (2.4)	13.30 (3.1)	0.744

When lipid parameters were compared according to PPAR alpha L162V genotypes, no statistically significant difference was found between genotypes (Table 6).

**Table 6:** Lipid parameters according to PPAR-alpha L162V genotypes IQR: interquartile range; LDL: low-density lipoprotein; HDL: high-density lipoprotein; VLDL: very-low-density lipoprotein * p-values are derived from the Kruskal-Wallis test

Parameters	PPAR-alfa L162V			p-values*
	L/L	L/V	V/V	
	Median (IQR)	Median (IQR)	Median (IQR)	
LDL	101.00 (35)	99.50 (45)	106.00 (73)	0.187
HDL	42.00 (9)	41.50 (15)	38.00 (14)	0.571
Cholesterol	171.00 (33)	169.00 (52)	164.00 (53)	0.443
Triglyceride	146.00 (67)	119.50 (70)	89.00 (47)	0.116
VLDL	28.00 (13)	24.00 (14)	18.00 (9)	0.110

To examine whether the PPAR alpha genotype influences the risk of ACS, carriers of the L and V-alleles were compared. V/V homozygous or L/V heterozyogus individuals showed a tendency towards a higher risk of ACS compared with homozygous carriers of the common L-allele, with an odds ratio of 1.96 (95% CI 1.08-3.55).

## Discussion

The important point in reducing the mortality and morbidity associated with CAD is to identify people at risk of CAD and to ensure their early protection. 15-19% of patients with CAD do not smoke or have diabetes mellitus, hypertension, or hyperlipidemia. However, 50% have at least one risk factor [18]. There are studies in the literature showing that PPAR gene polymorphism may be a genetic marker in the development of various cardiovascular diseases. However, these studies have found different results [18-21]. Therefore, this study aimed to evaluate the role of PPAR alpha and gamma gene polymorphisms in the development of the acute coronary syndrome.

Other studies have investigated acute coronary syndrome, CAD, and PPAR gamma C161T gene polymorphism [16,19,22]. However, these studies have yielded conflicting results. In some studies, the T/T allele was found to be protective in CAD patients, although other studies found that the T/T allele was a risk factor in the development of ACS [19,22,23]. In some studies, similar results have been obtained between patients and control groups [16,20,21]. These differences may be due to the relatively small size of patient groups or inclusion criteria. Chao et al. conducted a study in China with 146 patients under 50 years of age who were diagnosed with acute coronary syndrome and 146 volunteers in the control group [22]. In this study, the T/T genotype was found to be higher in acute coronary syndrome patients (13%) compared to the control group (5.5%). This difference was not detected in the C/C and C/T genotypes. Qian et al. investigated the C161T gene polymorphism in 137 patients with acute coronary syndrome, 281 patients with CHD, and 161 control group volunteers. In the comparison between the control group and the acute coronary syndrome group, the T allele was found more often in patients with acute coronary syndrome than in the C/C homozygous genotype (OR = 1.63, 95% CI 1.00-2.65, P = 0.048). No differences were found in the C161T polymorphism between the CHD patients and the control group [16]. Peng et al. found that the risk of chronic heart disease was lower in patients with the C/T genotype and higher in patients with the C/C genotype. They found no significant differences in terms of the T or C allele [20]. In our study, we found the C/C genotype in 26% and the C/T genotype in 74% of patients, while these genotypes were found in 93% and 7% of the control group, respectively. The frequency of the C allele was higher than the T allele in both the control group and the patient group, while the T allele was more common in the patient group (0.37) compared to the control group (0.03). This study also found that the C161T polymorphism increased the risk of acute coronary syndrome in line with previous studies [16,19,22]. Other studies have indicated that C161T polymorphism reduces the risk of CHD [23-25]. The effects of C161T polymorphism on CHD and acute coronary syndrome were found to be inversely related. This may be because the effects of PPAR gamma on pro-inflammatory mechanisms facilitate the plaque stability and inflammatory mechanisms that develop in acute coronary syndrome.

Furthermore, the incidence of PPAR alpha L162V polymorphism has been found to be approximately 6% in the Caucasian population [26]. In a study conducted in the United States, the incidence of L162V polymorphism was found to be 6.9% [17]. In our study, the PPAR alpha L162V polymorphism was found in 13% of the control group. Other studies in the literature have previously examined the PPAR alpha L162V polymorphism in patients with CHD and MI. In some studies, the L161V polymorphism was found to be a risk factor for CHD, although it was not found to be a risk factor in others [16, 27-30]. Reinhard et al. found no relationship between L162V polymorphism and MI [29]. Skoczynska et al. found that L162V polymorphism was higher in men with CHD than in the control group. They also found that the V allele was four times higher in patients with coronary atherosclerosis compared to the control group [27]. Sergeeva et al. investigated the L162V polymorphism in CHD patients and a control group. PPAR-alpha gene L162V genotype and V allele carriers were found to have a 2.21-fold increased risk of suffering from CHD [30]. Similarly, Qian et al. suggested that having a V allele in L162V polymorphisms increases the risk of CHD [16].

This study found that among the patients with acute coronary syndrome for whom PPAR alpha L162V polymorphism was found, 58% were carrying the L/L genotype, 23% the L/V genotype, and 19% the V/V genotype, and in the control group, 73% were carrying the L/L genotype and 27% the L/V genotype. The V allele was found to be statistically higher in patients with acute coronary syndrome. Different results in the literature may be due to the heterogeneity of the sample group and the inclusion criteria of the patients in the study group. For example, some studies included patients with CHD, while others included patients with coronary atherosclerosis. The inclusion criteria of patients with conditions such as stable angina, unstable angina, and unconfirmed vascular disease by coronary angiography may have affected the study results. These differences may also be due to the heterogeneous distribution of the clinical characteristics of the study groups. ACS physiopathology can also be affected by diabetes, hypertension, and cholesterol levels.

## Conclusions

CAD continues to be the most important cause of morbidity and mortality worldwide. This study aimed to investigate PPAR alpha and gamma gene polymorphisms in patients diagnosed with acute coronary syndrome. According to our study, the C161T polymorphism increases the risk of acute coronary syndrome, and the V allele is statistically higher in patients with acute coronary syndrome. Even if biochemical tests can reveal the risk of CAD, we believe that genetic information will be useful in predicting the risk of coronary artery disease in future generations. Thus, the incidence of coronary artery disease in individuals who are genetically possible candidates for coronary artery disease can be reduced in the future by controlling modifiable factors in the first years of life. The findings with respect to gene polymorphisms can contribute to future research on acute coronary syndrome.
